# ESTABLISHING THE EFFECTIVENESS OF THE ONLINE SAVVY FACILITATOR TRAINING PROGRAM

**DOI:** 10.1093/geroni/igad104.1017

**Published:** 2023-12-21

**Authors:** Carey Wexler Sherman, Kenneth Hepburn, John Hobday, Lai Reed

**Affiliations:** Savvy Systems, LLC, Minneapolis, Minnesota, United States; Emory University, Atlanta, Georgia, United States; Savvy Systems, LLC, Minneapolis, Minnesota, United States; Emory University, Atlanta, Georgia, United States

## Abstract

A total of 76 individuals took part in the revise training program we developed following prototype testing. These individuals were affiliated with 32 organizations in eight states. Each individual was scored on the observation/reflection provided at the end of each training session. Fifty-one percent of trainees received scores between 95-99% across all assessments; only 8.1% received summed scores less than 90%. In only 4 cases, was an individual required to repeat the observation/reflection exercise. Fifty-six trainees responded to a post-training evaluation survey that sought their Likert scale assessment (1=Strongly Disagree; 5=Strongly Agree) of their experience with elements of the online program and their sense of preparedness to lead SC program. The clarity and completeness of the program, overall, received a 4.40 rating, and the ratings for individual segments ranged from 4.40 to 4.5. Trainees gave the quizzes and assessments a 4.40 rating as learning aids. Trainees rated their overall preparedness to lead the SCP at 4.1. Trainees appreciated that the course was self- paced. They indicated particular benefit from the embedded videos, trainer manual, and the interactive materials and quizzes.

